# Supplementary figures and images for: EPHA5 regulates antifungal innate immunity by phosphorylating EPHB2 and Dectin-1
Source: PLoS Pathog. 2025 Jun 9;21(6):e1013179. doi: 10.1371/journal.ppat.1013179 (PMC12176286; doi:10.1371/journal.ppat.1013179)

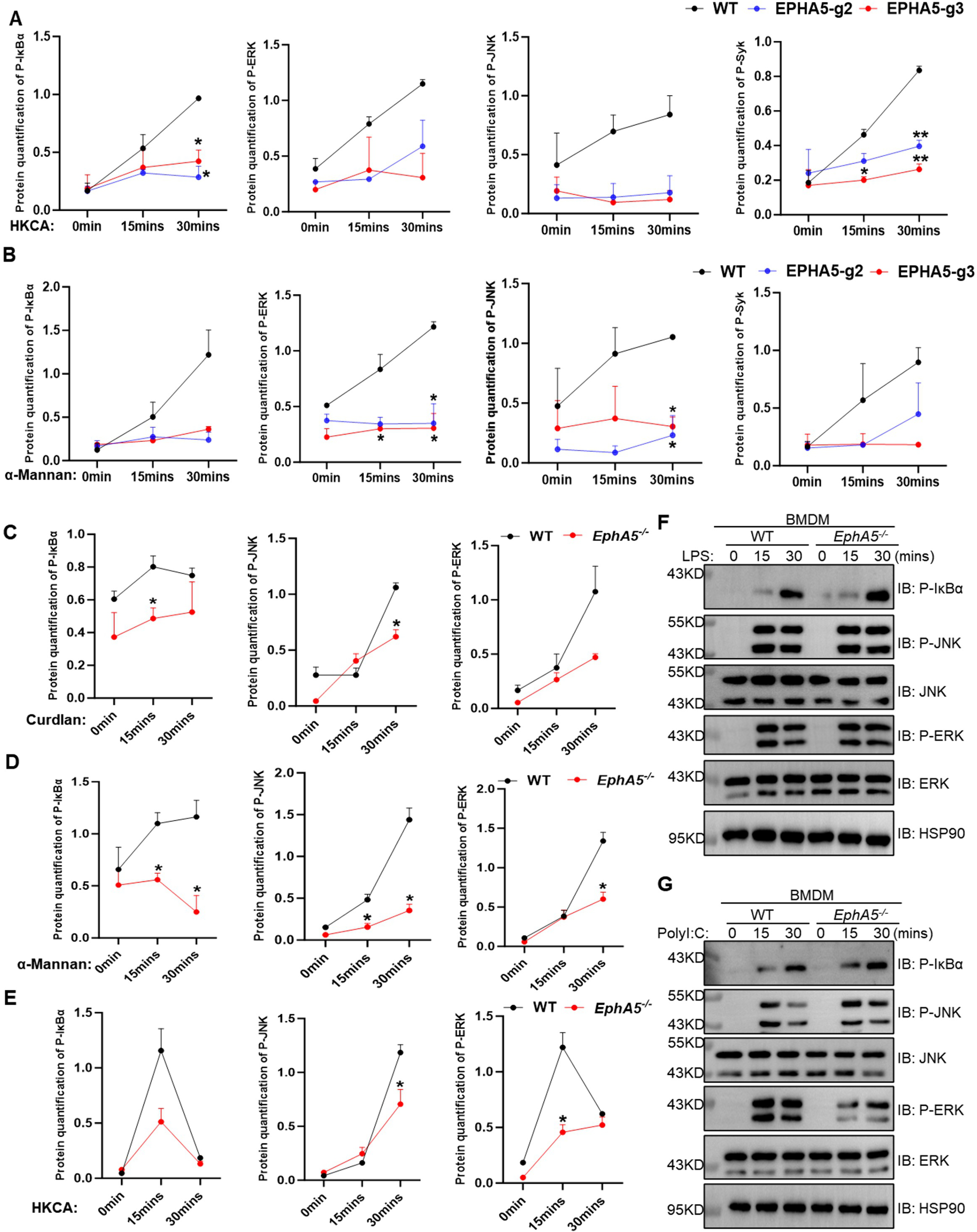

Supplement: S1 Fig — (A-B) Quantification of phosphorylated protein levels (P-IκBα, P-ERK, P-JNK, and P-Syk) on the basis of grayscale intensity analysis from Fig 1A and 1B was shown. (C-E) Quantification of phosphorylated proteins (P-IκBα, P-JNK, and P-ERK) from the grayscale intensity values in Fig 1C–1E was shown. (F-G) Western blot analysis of bone marrow-derived macrophages (BMDMs) from WT and EphA5-KO mice stimulated with LPS (20 ng/mL, F) or PolyI:C (100 ng/mL, G) for the indicated time points was shown to assess the activation of key signaling proteins. Data are presented as the mean ± standard error of the mean (S.E.M.) from biological replicates. Statistical significance was determined by a two-tailed unpaired t test (*P < 0.05; **P < 0.01; ***P < 0.001; ****P < 0.0001). (TIF) [file ppat.1013179.s001.tif]

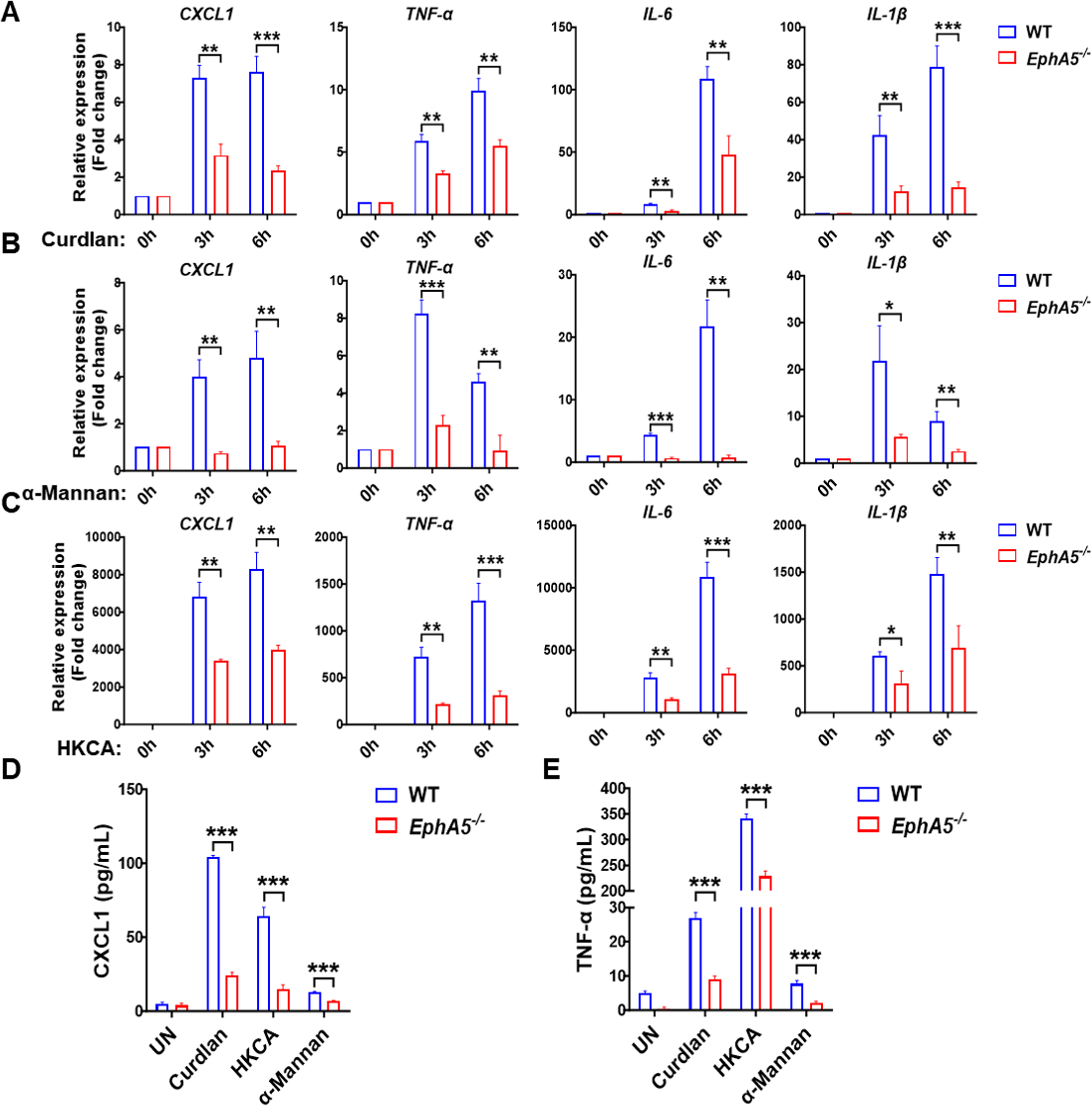

Supplement: S2 Fig — (A-C) RT‒qPCR analysis of CXCL1, TNF-α, IL-1β, and IL-6 expression in peritoneal macrophages from WT and EphA5-KO mice stimulated with Curdlan (100 μg/mL) (A), α-Mannan (100 μg/mL) (B), or HKCA (C) at the indicated time points was shown. (D-E) ELISA quantification of CXCL1 (D) and TNF-α (E) levels in supernatants from peritoneal macrophages stimulated with Curdlan (100 μg/mL), HKCA, or α-Mannan (100 μg/mL) for 24 hours was shown. The data are shown as the means ± S.E.M.s from biological replicates. Statistical significance was determined by a two-tailed unpaired t test (*P < 0.05; **P < 0.01; ***P < 0.001; ****P < 0.0001). The results are representative of three independent experiments. (TIF) [file ppat.1013179.s002.tif]

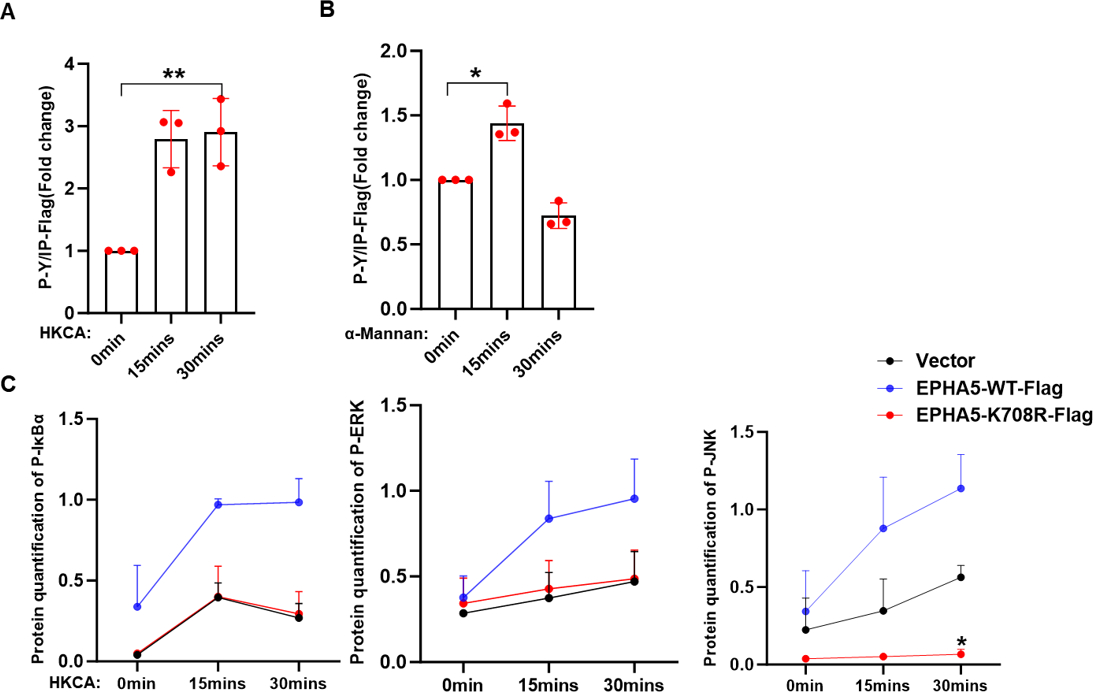

Supplement: S3 Fig — (A-B) Quantitative analysis of the grayscale intensity ratio of P-Y to IP-Flag in Fig 4A and 4B was shown. (C) Quantification of phosphorylated proteins (P-IκBα, P-ERK, and P-JNK) via grayscale intensity values from Fig 4C was shown. (TIF) [file ppat.1013179.s003.tif]

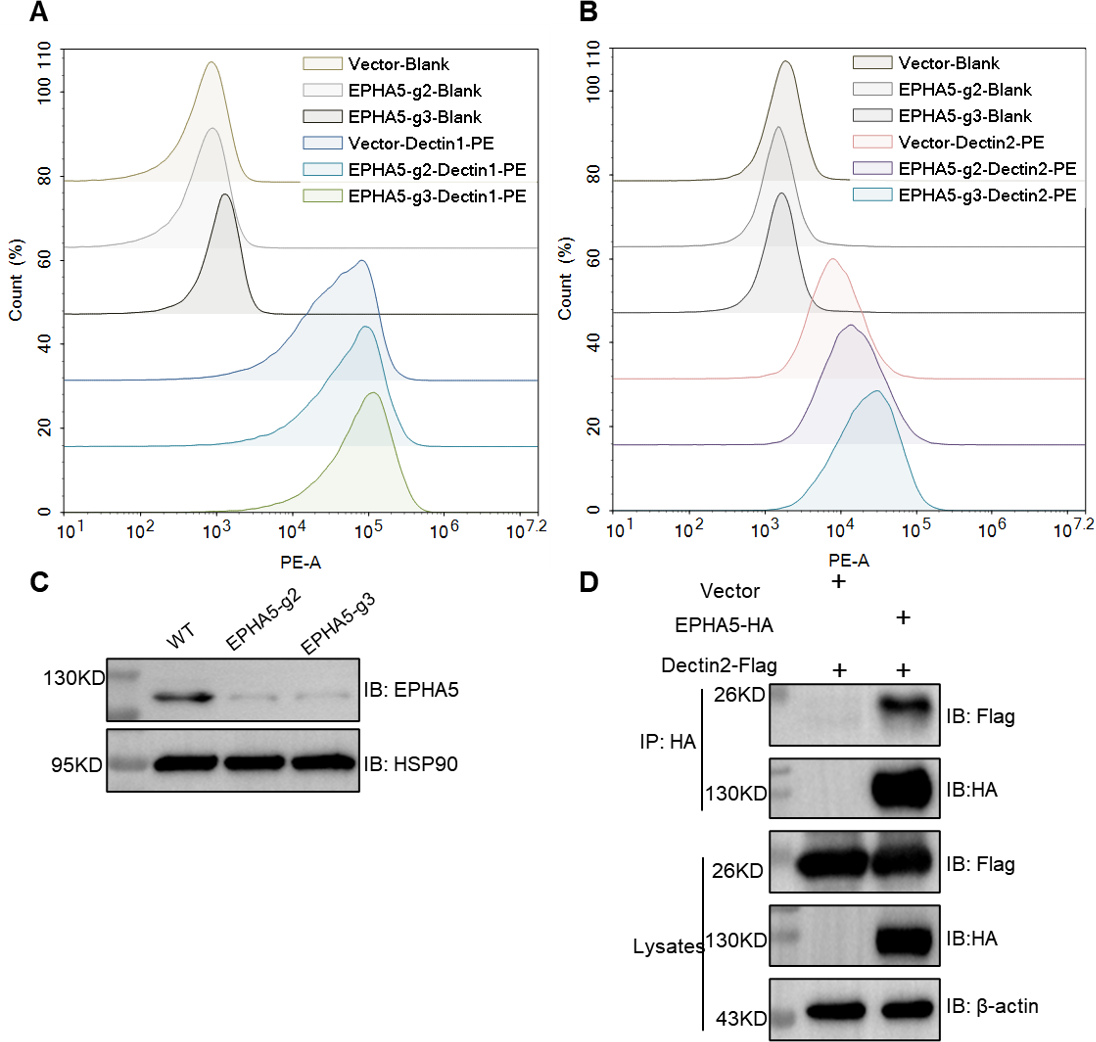

Supplement: S4 Fig — (A) Flow cytometry analysis of Dectin-1 and Dectin-2 expression in control (Vector) or EPHA5 knockdown THP-1 cells (EPHA5-g2 and EPHA5-g3) was shown. (B) Western blot validation of the EPHA5 knockout efficiency in THP-1 cells. (C) HEK293T cells were co-transfected with Dectin2-Flag and EPHA5-HA, followed by immunoprecipitation with an anti-HA antibody, and western blot analysis was shown for the indicated proteins. (TIF) [file ppat.1013179.s004.tif]

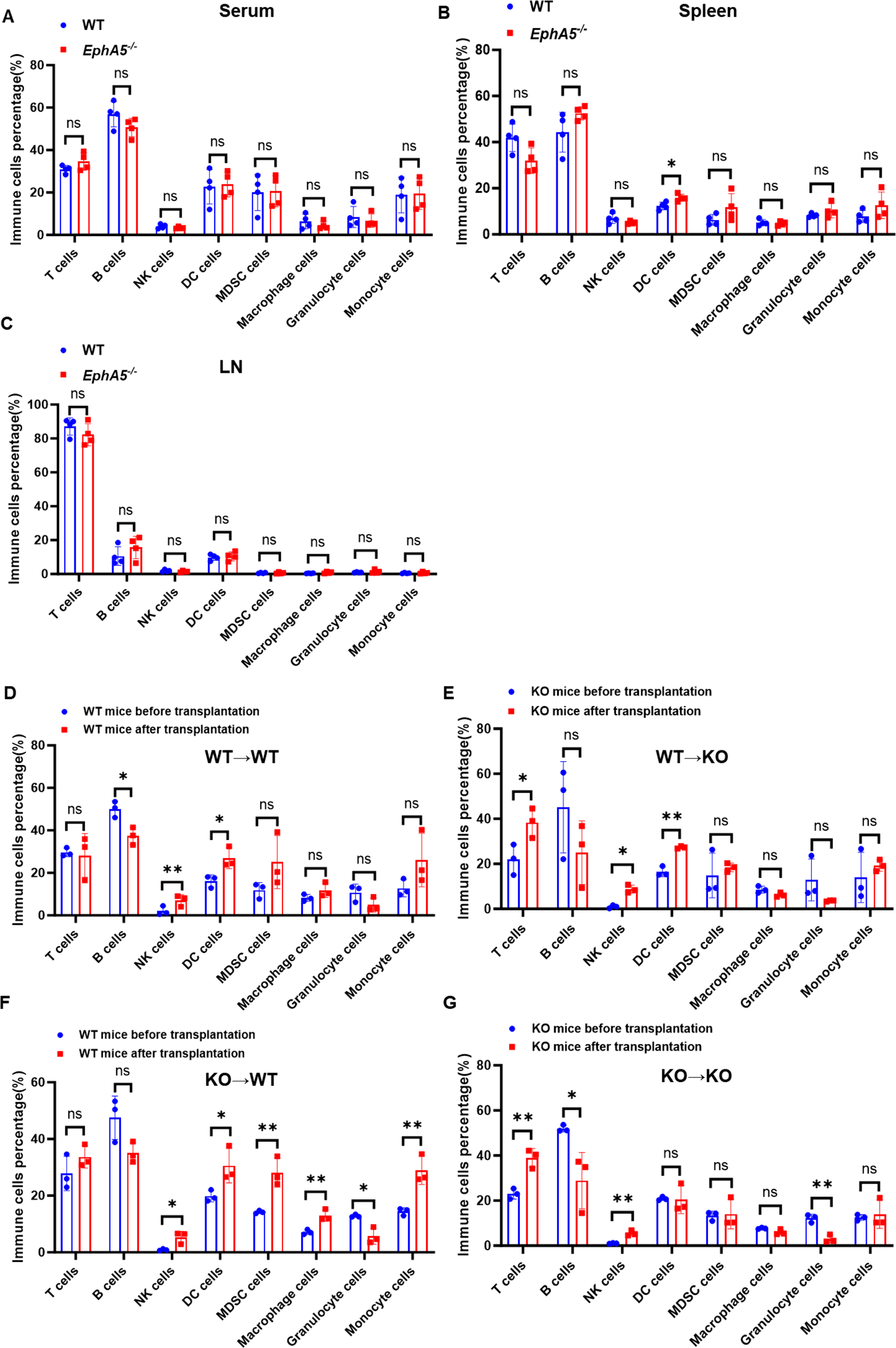

Supplement: S5 Fig — (A-C) Flow cytometric analysis of immune cell subsets in the serum, spleen and lymph nodes (LNs) of WT and EphA5 ⁻ / ⁻ mice was shown. The percentages of immune cells, including T cells, B cells, NK cells, dendritic cells (DCs), myeloid-derived suppressor cells (MDSCs), macrophages, granulocytes, and monocytes, were shown. Each dot represents an individual mouse (n = 4 per group). (E-H) Flow cytometric analysis of immune cell subsets in bone marrow chimeric mice following irradiation and reconstitution with WT or EphA5 ⁻ / ⁻ bone marrow was shown. The immune cell distribution in the serum of the recipient mice was assessed. Each dot represents an individual mouse (n = 5 per group). Statistical significance was determined by a two-tailed unpaired t test (ns, not significant; *P < 0.05; **P < 0.01; ***P < 0.001; ****P < 0.0001). (TIF) [file ppat.1013179.s005.tif]

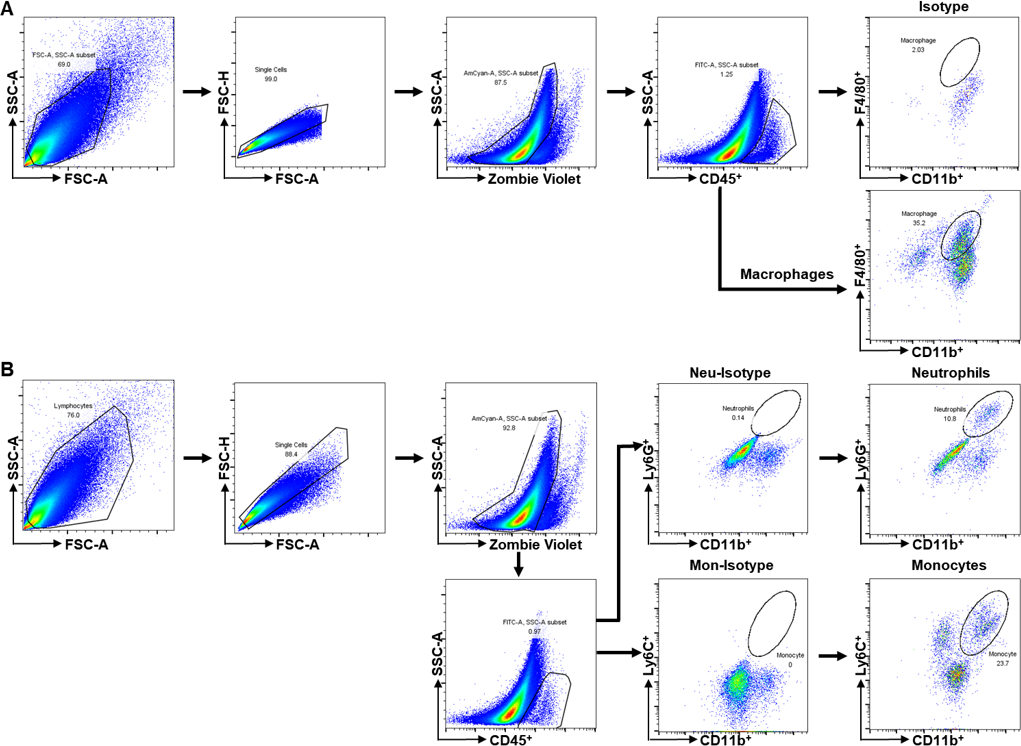

Supplement: S6 Fig — (A) The gating strategy for macrophages was shown: single cells were first gated on FSC-A and SSC-A, followed by the exclusion of dead cells via Zombie Violet staining. The immune cells were identified as CD45 ⁺ , and the macrophages were further gated as F4/80 ⁺ CD11b⁺ cells. (B) The gating strategy for granulocytes and monocytes was shown: single cells were gated on FSC-A and SSC-A, with live cells identified by Zombie Violet staining. The immune cells were gated as CD45 ⁺ , with granulocytes defined as Ly6G⁺CD11b⁺ and monocytes as Ly6C⁺CD11b ⁺ . (TIF) [file ppat.1013179.s006.tif]
