# Supplementary material for: EPHA5 regulates antifungal innate immunity by phosphorylating EPHB2 and Dectin-1
Source: PLoS Pathog. 2025 Jun 9;21(6):e1013179. doi: 10.1371/journal.ppat.1013179 (PMC12176286; doi:10.1371/journal.ppat.1013179)
Supplement: Attachment — Submitted filename: Response to Reviewers.docx [file ppat.1013179.s009.docx]

**Response to Reviewers**

We are grateful to the reviewers for their insightful comments and suggestions, which have been instrumental in enhancing the quality of our manuscript. Below, we provide point-by-point responses to the reviewers' concerns, indicating how each issue has been addressed in the revised manuscript.

**Comments from reviewer(s): Reviewer #1**

**Part I - Summary**

The manuscript “EPHA5 regulates antifungal innate immunity by phosphorylating EPHB2 and Dectin-1” investigated the function of EphA5 during antifungal immunity. The authors found that EPHA5 plays a critical role in antifungal immunity by phosphorylating EPHB2 and Dectin-1 after fungal infection. The authors used overexpression of WT and kinase dead receptor variants in different cell line to show physical interaction and receptor crosstalk of EphA5, EphB2, and dectin-1 upon fungal stimulation. Finally, the authors used Epha5 KO mice, as well as chimeric mice with deficiency in the hematopoietic compartment, to show that EphA5 is required for protection against lethal candidiasis. The manuscript is well written. However, some questions remain.

We thank the reviewer for these comments.

**Part II – Major Issues: Key Experiments Required for Acceptance**

1. Fig1: It is a bit surprising that in their Epha5 KO CRISPR THP1 cell lines a slight band within an immunoblot remains (same for the BMDMs). This needs clarification or probing with a different antibody. Also, the authors should check if EphA5 deletion reduces cell surface expression of CLRs including dectin-1/2/3.

We thank the reviewer for the comments and suggestions. For Fig 1A-B, EPHA5-gRNA-infected THP-1 cells were generated via CRISPR/Cas9, and GFP-positive cells (EPHA5-gRNA plasmid-infected cells) were pooled to perform the experiments. Therefore, the EPHA5-g2/g3 cells were not completely EPHA5 knockout cells and may be included with wild-type cells, which is the reason that a slight EPHA5 band remained. This strategy will be able to avoid single-clone effects. We also replaced the original results with the data exhibiting greater differences, leading to the revision of **Fig 1A-D**. Specifically, the previous **Fig 1C-E** has been substituted with the current **Fig 1F-H**, whereas the previous **Fig 1F-H** is now presented as **Fig 1C-E**. The grayscale intensity analyses corresponding to **Fig 1A-E** have been put to **S1A-E Fig** respectively.

In accordance with the reviewer’s suggestion, we investigated the surface expression of Dectin-1 and Dectin-2 in EPHA5-gRNA-knockdown THP-1 cells compared with control cells via flow cytometry analysis. Our findings indicate that the surface expression of Dectin-1 and Dectin-2 remains unaffected in EPHA5-Grna-infected cells compared with control cells. These results are shown in **S4A-C Fig** of the revised manuscript.

2. Fig6: Unclear why data from weight loss and survival are shown from different Candida inoculum. Please add inoculum for kidney fungal burden in the legend not only in the text. Please provide total numbers of infiltrating neutrophils, monocytes, and macrophages rather than only percentages.

We thank the reviewer for the comments and suggestions. Weight loss and survival experiments were conducted separately. In the weight loss experiment, the mice were inoculated with 2 × 10⁵ CFU of *C. albicans* SC5314 per mouse, whereas in the survival experiment, the inoculum size was increased to 4 × 10⁵ CFU of *C. albicans* SC5314 per mouse. The inoculation dose for the renal fungal burden assessment is shown in the legend of **Fig 7C**, where WT and *EphA5⁻/⁻* mice were infected with 2 × 10⁵ CFU of *C. albicans* per mouse for 48 hours. These details are provided on **page 15, lines 331-333**. Furthermore, the results of the statistical analysis of the neutrophil, monocyte, and macrophage counts are presented in **Fig 7H-J**.

3. -For the bone marrow chimeric mice experiments: the authors need to provide data of archived chimerism. Also, missing groups are KO->KO and WT->KO. Is it possible that EphA5 has a function on radio-resistant cells during antifungal immunity as well? As seen for EphA2 (PMID: 36138043)

We thank the reviewer for the comments and suggestions. We conducted a comprehensive analysis of myeloid cells in mice both before and after transplantation. Using advanced experimental methodologies, we have meticulously examined the cellular composition and functional integrity of myeloid cells before and after irradiation, and our findings indicate that there are no significant defects in the cells after irradiation compared with no irradiation. These data are comprehensively presented in **S5D-G Fig** of the revised manuscript.

Furthermore, to address the specific concerns raised regarding the experimental groups, we incorporated additional experimental groups, namely, KO→KO and WT→KO, into the data. The WT→WT and WT→KO groups presented similar survival outcomes, and the KO→WT and KO→KO groups presented comparable survival outcomes（the difference didn't reach significance）, which indicates that hematopoietic cells expressing EPHA5 play important roles in the antifungal immune response. The data derived from these experiments are meticulously documented and illustrated in **Fig 7G**.

**Part III – Minor Issues: Editorial and Data Presentation Modifications**

1. The Candida glabrata/ auris data feel misplaced this late in the manuscript and should be integrated in earlier figures/parts.

We thank the reviewer for the comments and suggestions. The data pertaining to *Candida glabrata* and *Candida auris*, which were previously presented in **S2 Fig**, have now been repositioned in the revised manuscript as **Fig 2**. Additionally, an introductory description emphasizing the clinical relevance of these Candida species has been incorporated into the Introduction of the revised manuscript, specifically on **page 3, lines 33–37**.

**Comments from reviewer(s): Reviewer #2**

**Part I - Summary**

The manuscript by Gao et al describes the novel activation of EPHB2 and Dectin-1 by EPHA5 and the important functional consequences of this activation during fungal immunity. The authors provide strong evidence for EPHA5 phosphorylating dectin-1 at tyr 15 which promotes the efficient recruitment of Syk and downstream signalling. The authors further strengthen their study using EPHA5 deficient in vivo models. Overall, the results are very interesting and provide insight into a novel mechanism of CLR biology that has important consequences. However, the authors suggest EPHA5 has effect beyond Dectin-1 but do not investigate this. Please find my comments for the authors below.

We thank the reviewer for these comments.

**Part II – Major Issues: Key Experiments Required for Acceptance**

1. The authors use curdlan (Dectin-1), a-mannan (Dectin-2) and heat killed candida (likely multiple CLR ligands) throughout the manuscript. The authors show that EPHA5 mediates signalling in response to all three CLR ligands. Like most CLRs, Dectin-2 does not contain an itam domain and does not require tyr15 phosphorylation but instead associates with FcRg for signalling. The authors should clarify why they used a-mannan and discuss the implications of EPHA5 regulating broader CLR signalling.

We appreciate the reviewer’s comments and suggestions, and we discussed the implications of EPHA5 in regulating broader CLR signaling in the discussion section of the revised manuscript on **pages 17, line383 to page 18, line403**.

2. The discussion requires extensive development to appropriately place the findings of this study within our current understanding of CLR signalling and anti-fungal immunity.

We appreciate the reviewer’s comments and suggestions, and we revised the discussion section in the revised manuscript on **page 17, line375 to page 18,line403.**

**Part III – Minor Issues: Editorial and Data Presentation Modifications**

1) The flow cytometry in figure 6H and 6I is not clear.

a) The authors should add their gating strategies as a supplementary figure.

b) I am unconvinced by the f4/80 staining, the authors should include f4/80 isotype staining to better gate on f4/80+ cells.

c) Figure 6I likely contains neutrophils and monocytes, did the authors use Ly6G to remove neutrophils? This should be shown.

We thank the reviewer for the comments and suggestions. In response, we have made the following revisions to address the concerns raised and enhance the clarity and rigor of our manuscript:

a) To improve the clarity of the flow cytometry analysis presented in **Fig 6H-I**, we have added detailed gating strategies to **S6 Fig**. This figure provides a step-by-step illustration of the gating process used to identify macrophages (**S6A Fig**), neutrophils (**S6B Fig**), and monocytes (**S6B Fig**), thereby enhancing the transparency and reproducibility of our analysis.

b) To address concerns regarding the specificity of F4/80 staining, we included isotype control staining for F4/80 in **S6 Fig**. These control data confirm the specificity of F4/80^+^ cell population identification and validate the reliability of the gating strategy employed.

c) We appreciate the reviewer’s suggestion regarding the potential inclusion of neutrophils in **Fig 6I**. Our gating strategy, now detailed in **S6 Fig**, is based on well-established protocols (PMID: 35079145; PMID: 38166150). To further clarify the contribution of Ly6G^+^ neutrophils, we performed additional analyses to evaluate both their relative proportions and absolute numbers of Ly6G^+^ neutrophils. Please find the data in **Fig 7J** of the revised manuscript.

2) The methods state fungal burden was assessed after 5 days but the manuscript says 48 hours.

We thank the reviewer for this comment. We have revised the Materials and methods to correct this inconsistency. Specifically, we have updated the text on **Page 24, Lines 542-543** of the manuscript to state, “For fungal burden assessment, the mice were sacrificed 48 h post infection”.

**Comments from reviewer(s): Reviewer #3**

**Part I - Summary**

The manuscript by Gao et al. demonstrates the mechanistic role of EPHA-5 in host defense against C. albicans. Coupled with in vivo animal data, immunological data, and biochemistry, this group defines a non-redundant role for EPHA-5 to serve as a key protein to mediate phosphorylation of EPHB2 and Dectin-1. While there is plenty of data packed into this manuscript, there needs some additional attention to important controls and additional quantitation of biochemical data. These additional data would greatly strengthen the rigor of the stated conclusions.

We thank the reviewer for these comments.

**Part II – Major Issues: Key Experiments Required for Acceptance**

1. Introduction Line 10 – I understand that the authors are trying to indicate that antimicrobial resistance is the driving force. I would hesitate to use the word “misuse” as it implies intent. I don’t think that is what the authors intend.

We thank the reviewer for this suggestion. We agree that the term "misuse" could imply intent. To address this concern, we have updated the text on **Page 3, Lines 37-38** of the manuscript to state, “Despite progress in antifungal drug development, the efficacy of current therapies is increasingly undermined by increasing resistance and prolonged antifungal exposure”.

2. Results Line 11- Do you mean Fig 1C-E instead of Fig1A-B.

We thank the reviewer for this suggestion. We have revised the text to explicitly specify the cell type being referenced. The revised sentence is now on **Page 5, Lines 85-87**: “EPHA5 knockdown THP-1 cells displayed significantly reduced activation of the NF-κB and MAPK signaling pathways compared with those in THP1 WT cells (Fig 1A-B)”.

3. Please add catalog numbers to all antibodies and key reagents.

We thank the reviewer for the comments and suggestions. We meticulously compiled a comprehensive inventory of all the antibodies and key reagents utilized in this study. This information has been systematically organized and documented in the Materials and Methods section on **page 20, line445 to page 21, line 465**.

4. Which protease inhibitors were added for IP/Western experiments?

We thank the reviewer for the comments and suggestions. We incorporated a protease inhibitor cocktail (Roche, 11873580001) and phosphatase inhibitors (Roche, 4906845001) into our immunoprecipitation (IP) and Western blot experiments. This methodological detail has been meticulously documented in the Materials and Methods section of the manuscript, specifically on **page 22, lines 494-495.**

5. E;T should be used in place of MOI when heat-killed organisms are used. As they are dead, there is no infection.

We thank the reviewer for the comments and suggestions. In the manuscript, we have revised the terminology to replace "MOI" with "E; T" when referring to experiments involving heat-killed organisms.

6. Adjust spacing to Fig 1A, B, F, G, and H to group the whole protein western blot with the phosphorylated protein interrogated.

We thank the reviewer for the comments and suggestions. In the revised manuscript, the original data for the phosphorylated protein (previously presented in Fig 1F–H) has been repositioned to the current **Fig 1C–E**, while the original data for RT-qPCR (previously in Fig 1C–E) has been moved to the current **Fig 1F–H**.

7. What does g2, g3 mean in figure 1?

We thank the reviewer for this comment. In the figure legends for Fig 1A–B, g2 and g3 represent two distinct pools of EPHA5-gRNA-knockdown THP-1 cells (the sequences of EPHA5-gRNA in g2-RNA and g3-RNA differ). The revised statement has been updated to: “(A–B) WT THP-1 cells and EPHA5 knockdown (EPHA5-g2 or EPHA5-g3) THP-1 cells were stimulated with heat-inactivated *C. albicans* (HKCA, E; T = 2) (A) or α-Mannan (100 μg/mL) (B) for the indicated times, followed by western blot analysis to determine the levels of the indicated proteins,” and is now located on **page5, lines 92–93**.

8. Quantitation of blots for all would be helpful, especially, Figures 1F, 1G, 1H, 3D, 3H.

We thank the reviewer for their comments and suggestions. To address the concerns, we have performed grayscale intensity analyses for the protein bands depicted in the current Fig 1A–E, 4A–B, 4D, and 4H–I. The corresponding quantification data can be found in **S1A–E Fig** (for Fig 1A–E)**, S3A-B Fig** (for Fig 4A–B), **S3C Fig** (for Fig 4D)**, and Fig 4J** (for Fig 4D) of the revised manuscript, respectively.

9. Is Figure 4 done with g1 or g2 THP-1 KO cells?

We thank the reviewer for this comment. In the figure legend for Fig 4A, we explicitly state that EPHA5-g2-RNA-knockdown THP-1 cells were reconstituted with WT EPHA5 (EPHA5-WT). The revised statement has been updated to: “EPHA5 knockdown (EPHA5-g2) THP-1 cells were reconstituted with WT EPHA5 (EPHA5-WT)” and is now located on **page 9, lines 169-170**.

**Part III – Minor Issues: Editorial and Data Presentation Modifications**

1. The use of fungal ligands demonstrates differences between WT and KO cells. However, since EPHA5 has not been previously implicated in immune responses, it would be prudent to use a stimulant that does not engage Dectin1 or Dectin 2 (e.g. LPS - engaging TLR4). The expectation is that this activation would still be intact in KO cells and comparable to WT cells. This would increase the rigor of these conclusions.

We thank the reviewer for their comments and suggestions. To address this critical point, we conducted additional experiments in which both WT and EphA5-KO BMDMs were stimulated with the canonical TLR ligands LPS (a TLR4 agonist) and PolyI:C (a TLR3 agonist). As shown in **S1F-G** **Fig**, *EphA5* deficiency does not compromise TLR signaling, excluding nonspecific developmental or functional defects in KO macrophages.

2. Some of the differences in biochemistry are subtle. Quantification of these blots would go a long way to establish significance, as stated by the authors. Significance indicates a mathematical difference, but these data are not provided.

We thank the reviewer for the comments and suggestions. We performed grayscale intensity analyses for the protein bands depicted in the current **Fig 1A-E, 4A-B, 4D, 4H-I.** The corresponding quantification data can be found in **S1A–E Fig** (for Fig 1A–E)**, S3A-B Fig** (for Fig 4A–B), **S3C Fig** (for Fig 4D)**, and Fig 4J** (for Fig 4D) of the revised manuscript, respectively.

3. Since the KO mice have not been examined for any immunological defects, can the authors either cite a paper or provide data that these mice do not have any gross immunological defects that could skew the interpretation (i.e. number of circulating neutrophils, changes in the spleen, lymph nodes, etc.).

We thank the reviewer for this comment. To determine whether the increased susceptibility of the *EphA5*-KO mice was due to underlying immunodeficiency, we examined the distributions of major immune cell populations in the peripheral blood, lymph nodes, and spleen under steady-state conditions. Flow cytometric analysis revealed no significant differences in the frequencies of key immune cell subsets, including T cells, B cells, macrophages, and neutrophils, between WT and *EphA5*-KO mice, and there was no significant difference in terms of different cell types between WT and *EphA5*-KO mice (**S5A‒C Fig**).

4. How do the authors reconcile the TNF data shown in Figure 3A with S1C,E?

We thank the reviewer for the comments and suggestions. The previous Fig 3A has been renumbered to Fig 4A, and the previous S1C Fig and S1E Fig have been renumbered to S2C Fig and S2E Fig, respectively. The revised data in Fig 4A illustrate the time-dependent increase in EPHA5 tyrosine phosphorylation following stimulation with heat-killed *Candida albicans* (HKCA). TNF-α is an important cytokine secreted by monocytes/macrophages after fungi infection (PMID: [36377664](https://pubmed.ncbi.nlm.nih.gov/36377664/); PMID: 35079145) .The data presented in **S2C Fig** depict the changes in TNF-α mRNA levels after peritoneal macrophages were stimulated with HKCA, and **S2E Fig** shows the alterations in TNF-α protein levels in the culture supernatant of peritoneal macrophages following stimulation with curdlan, HKCA, or α-mannan. These revisions ensure a more accurate and organized presentation of the experimental findings, reflecting the dynamic changes in protein expression and phosphorylation in response to fungal stimuli.

5. The discussion misses the opportunity to discuss these findings in the broader context and instead focuses on potential use in therapeutics. I would love to hear their thoughts on possible ligands, for example.

We thank the reviewer for the comments and suggestions. We revised the discussion section of the manuscript, especially discussed the upstream signal of EPHA5 after fungal stimulation on **page 18, line 404 to page 19, line 419**.

We believe that the revised manuscript has significantly improved in clarity and rigor. We appreciate the opportunity to resubmit this manuscript and remain open to any further adjustments.
